# Overview of a User-Centered, Mixed-Methods Process for Designing Interconnected and Focused Mobile Applications on Patient Care Environment (InterFACE): Augmented-Reality Decision Support System for Pediatric Resuscitation

**DOI:** 10.2196/78144

**Published:** 2026-02-13

**Authors:** Frederic Ehrler, Ana Rajic, Alexandre De Masi, Sharleen Olanka, Marco Generelli, Jennifer Davidson, Yiqun Lin, Kangsoo Kim, Pierre-Louis Rebours, Marc Ibrahim, Donovan Duncan, Ryan Kang, Sergio Manzano, Adam Cheng, Johan N Siebert

**Affiliations:** 1 Direction of information science, University Hospital of Geneva, Gabrielle Perret Gentil 4, Geneva, 1203, Switzerland, 41 765327838; 2 Faculty of Medicine, University of Geneva, Geneva, Switzerland; 3 Educational Technologies and Learning Sciences (TECFA), Faculty of Psychology and Educational Sciences, University of Geneva, Geneva, Switzerland; 4 KidSIM-ASPIRE Simulation Research Program, Alberta Children's Hospital, University of Calgary, Calgary, AB, Canada; 5 Departments of Pediatrics and Emergency Medicine, Cumming School of Medicine, University of Calgary, Calgary, AB, Canada; 6 Department of Electrical and Software Engineering, Schulich School of Engineering, University of Calgary, Calgary, AB, Canada; 7 Pediatric Intensive Care Unit, Alberta Children's Hospital, University of Calgary, Calgary, AB, Canada; 8 Department of Pediatric Emergency Medicine, Geneva Children's Hospital, University Hospital of Geneva, Geneva, Switzerland

**Keywords:** implementation, rescucitation, augmented reality, pediatric, team coordination, digital cognitive aids, situational awareness, user-centered design

## Abstract

**Background:**

Pediatric cardiopulmonary resuscitation (CPR) is a highly complex and time-critical process that demands precise team coordination and strict adherence to pediatric advanced life support (PALS) guidelines. In real-world practice, adherence often deteriorates due to cognitive overload, fragmented communication, and disruption of information flow under stress. Although digital cognitive aids have shown potential to improve adherence, existing tools are often limited to single tasks, lack team-wide integration, or fail to adapt in real time to dynamic clinical environments.

**Objective:**

This study aimed to design and evaluate InterFACE (Interconnected and Focused Mobile Applications on Patient Care Environment), an integrated, augmented reality (AR)–enabled digital health system developed to support real-time PALS adherence and enhance team coordination during pediatric resuscitation.

**Methods:**

A structured, mixed methods, user-centered design process was used. Persona development and spatial analysis characterized the needs and positions of key resuscitation roles. A 3-round Delphi process with experts identified critical information elements for display. Iterative user experience (UX) prototyping was performed, followed by simulation-based evaluations of three system components: (1) TeamScreen, a wall-mounted team display providing a shared overview of the resuscitation process; (2) Guiding Pad (developed by Pierre Louis Rebours and Marc Ibrahim), a tablet-based app for documentation and algorithm navigation; and (3) AR head-mounted displays (HMDs) for team leaders and medication nurses, delivering role-specific, context-aware guidance. Usability was assessed with standardized instruments, including the System Usability Scale (SUS), Technology Acceptance Model (TAM), and User Experience Questionnaire (UEQ).

**Results:**

The Delphi study achieved consensus on 20 core information elements, distributed across the 3 interfaces. Usability testing demonstrated high acceptance across all modalities. The Guiding Pad supported effective navigation of resuscitation algorithms with a 78%-100% task completion rate. The TeamScreen achieved an overall task success rate of 81%, improving situational awareness despite some confusion in high-density regions. AR HMDs received favorable evaluations, with SUS scores rated “Good” to “Excellent,” and UEQ ratings indicating high intuitiveness, stimulation, and attractiveness. Participants consistently described InterFACE as intuitive, useful for real-time decision-making, and supportive of team synchronization. Reported challenges included interface complexity, incomplete integration with patient monitors, and potential cognitive load from simultaneous information streams.

**Conclusions:**

InterFACE represents a significant advancement in digital cognitive aids by combining shared displays, tablets, and AR guidance into a synchronized, role-specific ecosystem. The system shows promise in enhancing adherence to PALS, reducing cognitive load, and improving team coordination in simulated pediatric resuscitations. While results demonstrate strong usability and acceptance, further research is needed to evaluate clinical effectiveness in real-world settings, including randomized controlled trials, integration with hospital information systems via Fast Healthcare Interoperability Resources (FHIR) standards, and potential artificial intelligence–driven decision support to optimize adaptability and long-term skill retention.

## Introduction

Cardiopulmonary resuscitation (CPR) in pediatric and adult emergencies is a complex, time-sensitive process requiring precise team coordination. Adherence to pediatric advanced life support (PALS) guidelines, such as those from the American Heart Association (AHA) [1] and European Resuscitation Council [2], is crucial for optimizing CPR quality and patient outcomes [3]. Yet, the high-stress environment of CPR often disrupts guideline adherence due to cognitive overload, miscommunication, and fragmented information flow, impairing decision-making and delaying critical interventions.

Digital health technologies offer promising solutions to support resuscitation teams in maintaining guideline compliance and coordination. Previous research has investigated digital resuscitation tools like head-mounted displays (HMDs) [4], tablet-based guidance systems [5,6], and mobile apps to support caregivers, enhance adherence to resuscitation guidelines, and minimize drug preparation errors [7,8]. While these technologies have demonstrated potential benefits, they also reveal limitations in usability, team-wide information sharing, and real-time adaptability to dynamic clinical scenarios.

**Figure 1.**
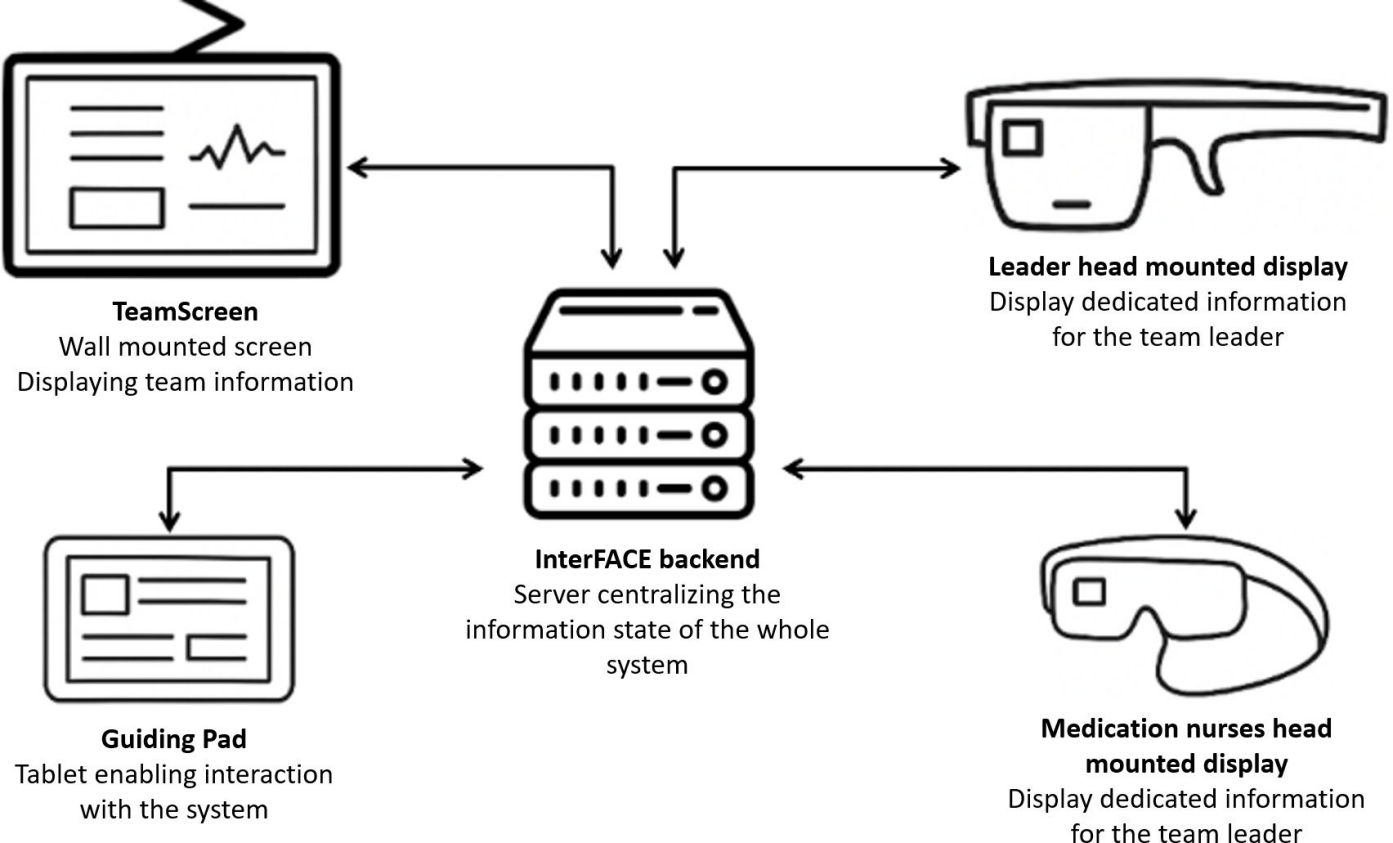
System architecture of InterFACE, illustrating the conceptual flow of information between the backend and user interfaces. InterFACE: Interconnected and Focused Mobile Applications on Patients Care Environment.

To address these limitations, we developed InterFACE (Interconnected and Focused Mobile Applications on Patients Care Environment), an integrated digital suite designed to enhance pediatric CPR performance through real-time, role-specific guidance and improved team coordination aligned with pediatric PALS algorithms. InterFACE comprises three core components: (1) a wall-mounted display delivering a dynamic, real-time resuscitation overview for the team; (2) a tablet app, operated by the documenting (script) nurse, enabling CPR algorithm navigation and task documentation; and (3) mixed reality (augmented reality, AR) HMDs, worn by the team leader and medication nurse, providing context and role-specific, interactive guidance (Figure 1). Unlike traditional AR, which overlays static digital data, InterFACE leverages AR technology to integrate spatially anchored, interactive virtual elements into the physical environment to enhance situational awareness and enable hands-free access to critical information. InterFACE aims to deliver accurate, context-specific information to each team member in real time, enhancing CPR efficiently through improved team alignment and PALS guidelines adherence.

Unlike existing solutions that typically address a single aspect of the resuscitation process, such as medication preparation or algorithm visualization, InterFACE proposes a comprehensive, multirole synchronized system integrating wall-mounted displays, tablets, and head-mounted AR interfaces. This integration aims to improve shared cognition, role-specific guidance, and real-time adaptability—key elements often missing in previous digital resuscitation tools.

This paper aimed to present the user-centered design process and formative usability evaluation of InterFACE, an integrated, AR-based decision support system for pediatric resuscitation. Rather than providing exhaustive detail on each methodological step, we offer a cohesive, high-level overview of the mixed methods process, including Delphi consensus, usability testing of AR interfaces, and informatics architecture, which guided the system’s development. Each component has been or will be the subject of dedicated manuscripts; here, our goal is to highlight how these complementary approaches were integrated into a coherent design framework for a complex clinical decision support system.

## Methods

### Overview

The InterFACE was designed and developed using a structured, multiphase, mixed methods approach, blending qualitative and quantitative methodologies to create a user-centered resuscitation decision support system aligned with clinical needs and real-world dynamics.

### Persona Development and Spatial Analysis

Personas representing key pediatric resuscitation roles (eg, team leader, medication nurse, proceduralist) were developed based on direct field observations and a series of semistructured interviews with clinical experts. Each persona captured role-specific responsibilities, cognitive workload, and critical information needs during high-stress resuscitation scenarios. The purpose of these personas was not to be directly evaluated but to serve as design scaffolds that informed the overall architecture of the InterFACE system. Specifically, they were used to: define role-specific interface requirements; guide how information was distributed across the 3 system components (tablet, AR head-mounted display, and team screen); ensure that each interface aligned with the cognitive workflow and situational demands of its corresponding clinical role.

In parallel, a spatial analysis was conducted within the actual resuscitation environment. This consisted primarily of mapping the typical positions and movement patterns of team members, assigning them to defined spatial zones (eg, head of bed, medication zone, and documentation area). This approach allowed us to identify where each team member typically stands or moves, and how they interact during various phases of care. Combining persona insights with spatial analysis ensured that digital information was presented in a way that matched both the cognitive and physical workflows of pediatric resuscitation.

### Expert Consensus Process: Delphi Study

A 3-round Delphi study established essential system information elements via expert consensus.

#### Round 1: Item Generation

Resuscitation experts from our research team reviewed 4 personas (team leader, scribe, medication nurse, and other team members) and current PALS guidelines, identifying critical real-time resuscitation data elements via open-ended responses. These data elements, such as heart rate, oxygen saturation, time of drug administration, and the next expected algorithm step, were thematically analyzed to generate an initial item list representing the minimal critical information required for decision-making and situational awareness during pediatric resuscitation.

#### Round 2: Prioritization

Each item was rated for importance on a pediatric 5-point Likert scale by resuscitation experts (emergency doctors and nurses) from Geneva Children’s Hospital and Alberta Children’s Hospital. Items with mean scores <3 were excluded; those with high variability (SD>1.5) were discussed further for agreement to decide their inclusion. Qualitative feedback refined the list.

#### Round 3: Final Consensus

Experts finalized the list, with ≥80% agreement defining inclusion.

### Iterative User Experience (UX) Design

A human-centered process ensured usability and workflow alignment.

#### Static Prototyping

Initial prototypes visualized layout, role-specific interfaces, information architecture, and functionality, undergoing internal testing for readability, functionality, and clarity.

#### Dynamic Prototyping

Refined static prototypes added interactivity, minimizing cognitive workload and aligning with resuscitation constraints, validated through iterative testing.

### Simulation-Based Evaluation

The usability and feasibility of the InterFACE suite was evaluated through simulated resuscitation scenarios designed to mimic real-world pediatric cardiopulmonary arrests. Each system component was independently tested to assess its performance, usability, and integration into clinical workflows. For the wall-mounted display, namely TeamScreen, participants completed 21 information-retrieval tasks, with performance gaged by task success rates, completion times, and score from the Post-Study System Usability Questionnaire (PSSUQ) [9]. The tablet underwent testing with 27 interaction tasks, where effectiveness was measured through task success, time-to-completion, and PSSUQ scores, ensuring the app’s alignment with dynamic resuscitation demands. The AR HMDs were assessed following a simulated CPR scenario, during which the team leader and medication nurse received guidance via Microsoft HoloLens 2. Usability was evaluated using the System Usability Scale (SUS), a 10-item questionnaire measuring perceived usability (effectiveness, efficiency, satisfaction) [10]; Technology Acceptance Model (TAM), a 12-item questionnaire assessing perceived usefulness and ease of use [11]; and User Experience Questionnaire (UEQ), a 26-item questionnaire evaluating 6 user experience dimensions [12] to capture both practical and experiential feedback. This stepwise evaluation provided comprehensive insights into each component’s strengths and areas for refinement, prioritizing clarity, accessibility, and real-time utility in high-pressure settings.

### Informatics Development and Integration

Following the usability evaluations, informatics development synthesized insights from previous testing phases into a cohesive final system. This process emphasized user needs, cognitive ergonomics, and compatibility with clinical workflows, achieved through iterative refinement rather than a single overhaul. By addressing feedback on usability and functionality, the development phase ensured that InterFACE seamlessly integrated into resuscitation practices, balancing technical sophistication with practical application. The result was a system designed to minimize cognitive workload while maximizing support for pediatric CPR teams, ready for broader validation in future studies.

### Ethical Considerations

This study did not involve patients, patient representatives, or the collection or use of identifiable personal health data. All study activities were limited to health care professionals participating in system design workshops, semi-structured interviews, and simulation-based usability evaluations of the InterFACE system. According to institutional and regional policies at the Geneva University Hospitals (HUG) and the University of Calgary, studies that exclusively involve health care professionals for the purposes of system design, usability testing, and simulation-based evaluation, without involving patients or identifiable personal data are not classified as human subject research and are therefore exempt from formal review by a Research Ethics Committee / Institutional Review Board. All participants were informed about the study objectives, procedures, and voluntary nature of participation and provided informed consent prior to participation. Data collected during usability sessions and interviews were anonymized, and no personally identifiable information was recorded or retained. No financial compensation was provided.

## Results

### Persona Development and Spatial Analysis

**Figure 2.**
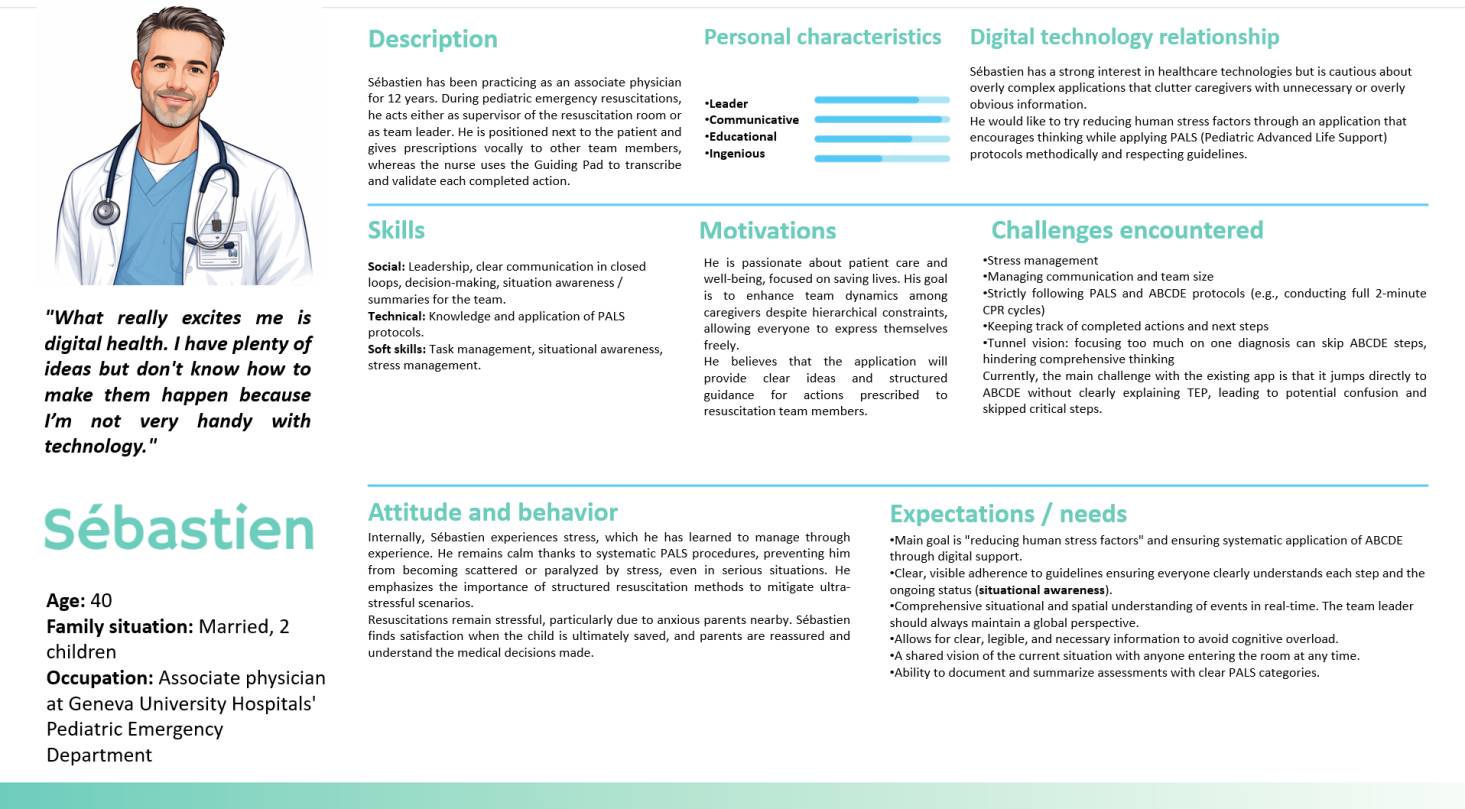
Persona profile of the team leader in pediatric resuscitation. This figure depicts a pediatric emergency physician with 12 years of experience as the team leader, detailing his responsibilities, competencies, motivations, challenges, and interaction with digital health technologies. The team leader plays a pivotal role in decision-making, coordination, communication, guideline adherence, and team performance under high-stress conditions.

To explore the behaviors, information needs, communication methods, and expectations of key actors involved in pediatric resuscitation, we conducted in-depth face-to-face interviews with 4 nurses and 4 physicians. These interviews offered valuable insights into team roles and interactions during resuscitation, identifying areas where effective information flow enhances performance and where communication gaps may hinder outcomes. A total of 4 distinct personas were developed, each representing a key resuscitation role. First, the team leader, a central coordinator, focuses on decision-making, information synthesis, and communication, requiring real-time, comprehensive patient data and clear channels, as detailed in the persona profile example in Figure 2.

**Figure 3.**
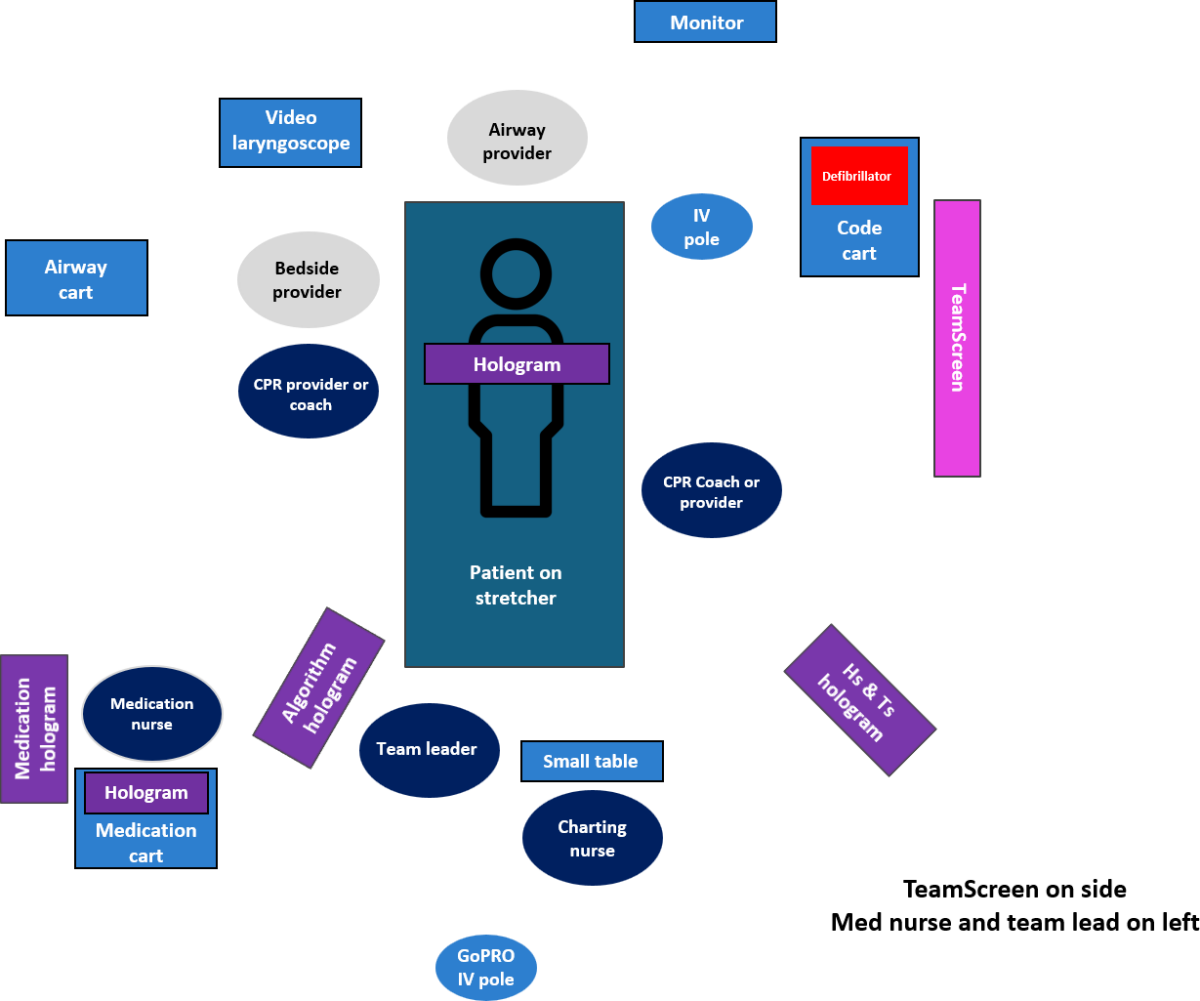
Cardiopulmonary resuscitation (CPR) provider placement in the study setting. Arrangement of team members and equipment around the manikin on a stretcher for effective resuscitation.

Second, the documenting nurse (scribe), responsible for real-time documentation, emphasized the need for a streamlined interface to record events while tracking verbal directives. Third, the medication nurse, tasked with drug preparation and administration, highlighted the importance of quick access to drug information and dosage guidelines through an error-minimizing interface. Fourth, the rest of the team, encompassing supporting roles with dynamic responsibilities, stressed the need for flexible communication tools to enable real-time collaboration, task coordination, and problem-solving. A spatial analysis, conducted through mapping and observation during two pediatric cardiac arrest simulated resuscitation scenarios, revealed consistent positioning patterns (Figure 3): the team leader was centrally located at the head of the bed for optimal oversight, the scribe was nearby for rapid documentation access, the medication nurse was positioned close to medication for swift access, and the rest of the team adapted dynamically to support primary roles.

### Delphi Study

The Delphi study’s first round generated 40 distinct information items, grouped into 8 thematic categories: Patient status (7 items), Change of status (6 items), Presentation of clinical task (4 items), Running timer (4 items), Drugs (5 items); CPR and defibrillation (4 items); PALS (7 items); and Teamwork (3 items; Figure 3). In the second round, a panel of 15 pediatric emergency nurses and 15 physicians rated these items on a 5-point Likert scale (Table 1). Strong consensus emerged for most items, except for displaying intravenous drug doses on the AR HMD where nurses and physicians held divergent views, prompting further discussion on optimal presentation. No significant differences were observed between groups, with both preferring the team-wide screen over AR HMD for real-time information, suggesting the TeamScreen’s role as the primary interface despite AR’s potential.

**Table 1.** Selection of 40 information items from the first round of the Delphi study. An item was considered selected if a participant rated it above 3 on the Likert scale. The selected items are organized into 8 thematic categories related to effective cardiopulmonary resuscitation (CPR) and pediatric advanced life support (PALS) management.

Thematic categories and selected items		Glasses		TeamScreen	
		MD^a^	RN^b^	MD	RN
Patient status, n (%)					
HR^c^		5 (33.33)	8 (53.33)	11 (73.33)	14 (93.33)
RR^d^		5 (33.33)	7 (46.67)	11 (73.33)	14 (93.33)
SpO_2_^e^		5 (33.33)	7 (46.67)	11 (73.33)	14 (93.33)
BP^f^		5 (33.33)	6 (40)	11 (73.33)	14 (93.33)
Temperature		3 (20)	2 (13.33)	9 (60)	9 (60)
EtCO_2_^g^		5 (33.33)	3 (20)	10 (66.67)	13 (86.67)
Pulse		6 (40)	8 (53.33)	10 (66.67)	14 (93.33)
Change of status, n (%)					
Cardiac rhythm		9 (60)	9 (60)	12 (80)	14 (93.33)
HR<60^h^		10 (66.67)	10 (66.67)	11 (73.33)	15 (100)
RR<8^i^		10 (66.67)	11 (73.33)	10 (66.67)	15 (100)
SpO_2_<80%		11 (73.33)	10 (66.67)	12 (80)	15 (100)
Loss of pulse		12 (80)	12 (80)	14 (93.33)	15 (100)
Return of pulse		9 (60)	12 (80)	11 (73.33)	15 (100)
Presentation of clinical tasks, n (%)					
Previous tasks		7 (46.67)	8 (53.33)	12 (80)	11 (73.33)
Current task		9 (60)	14 (93.33)	14 (93.33)	15 (100)
Next task		12 (80)	14 (93.33)	13 (86.67)	14 (93.33)
Running timer, n (%)					
CPR^j^ timer (2-minute countdown)		12 (80)	10 (66.67)	15 (100)	15 (100)
Pause in CPR timer (hands off time)		7 (46.67)	8 (53.33)	13 (86.67)	12 (80)
Epinephrine timer (4-minute countdown)		11 (73.33)	14 (93.33)	14 (93.33)	15 (100)
Overall event duration		5 (33.33)	7 (46.67)	13 (86.67)	13 (86.67)
Medication, n (%)					
IV/IO^k^ insertion location		0 (0)	0 (0)	4 (26.67)	5 (33.33)
Epinephrine dose		9 (60)	15 (100)	14 (93.33)	13 (86.67)
Amiodarone/ Lidocaine dose		7 (46.67)	13 (86.67)	12 (80)	14 (93.33)
Fluid bolus dose		4 (26.67)	12 (80)	9 (60)	13 (86.67)
Other medication dose		5 (33.33)	12 (80)	9 (60)	14 (93.33)
CPR and defibrillation, n (%)					
Initiate / resume CPR		9 (60)	8 (53.33)	15 (100)	13 (86.67)
Stop CPR / pulse check		9 (60)	7 (46.67)	15 (100)	14 (93.33)
Defibrillation timing (time to next shock)		10 (66.67)	7 (46.67)	12 (80)	14 (93.33)
Defibrillation dose		6 (40)	8 (53.33)	14 (93.33)	14 (93.33)
Advanced life support, n (%)					
Entire AHA^l^ clinical algorithm (whole flow diagram)		6 (40)	5 (33.33)	11 (73.33)	13 (86.67)
Advanced airway timing		4 (26.67)	7 (46.67)	7 (46.67)	9 (60)
ETT/LMA^m^ size		2 (13.33)	4 (26.67)	11 (73.33)	10 (66.67)
Check tube placement		2 (13.33)	2 (13.33)	5 (33.33)	7 (46.67)
Review reversible causes		9 (60)	3 (20)	13 (86.67)	12 (80)
Bedside echo		4 (26.67)	3 (20)	4 (26.67)	7 (46.67)
Page ECMO^n^ team		8 (53.33)	3 (20)	10 (66.67)	7 (46.67)
Teamwork, n (%)					
Role allocation		1 (6.67)	3 (20)	7 (46.67)	9 (60)
Case summary		2 (13.33)	6 (40)	10 (66.67)	10 (66.67)
Ask for team input		4 (26.67)	2 (13.33)	5 (33.33)	6 (40)
Mean (SD)		6.64 (2.76)	7.69 (3.19)	10.87 (2.22)	12.31 (2.36)

a MD: medical doctor.

b RN: registered nurse.

c HR: heart rate.

d RR: respiratory rate.

e SpO2: pulse oximetry.

f BP: blood pressure.

g EtCO2: end-tidal carbon dioxide.

h HR<60: heart rate less than 60.

i RR<8: respiratory rate less than 8.

j CPR: cardiopulmonary resuscitation.

k IV/IO: intravenous/intraosseous.

l AHA: American Heart Association.

m ETT/LMA: endotracheal tube/laryngeal mask airway.

n ECMO: extracorporeal membrane oxygenation.

The third round finalized a refined list, achieving over 80% consensus for inclusion. Although over two-thirds of experts strongly supported including patient vital signs, these were excluded due to technical constraints requiring monitor integration, which was beyond this version’s scope. A total of 7 items, primarily medication, timer, and tasks information, were selected for the medication nurses’ HMD, while the team leader’s HMD included these plus CPR and advanced life support data, totaling 20 items. The TeamScreen displayed the most items, adding 3 more—previous task, overall resuscitation duration, and ETT/LMA size. While physicians and nurses showed similar preferences, nurses favored broader visibility on the TeamScreen for monitoring patient status and rhythm changes, aligning with their assessment role, whereas physicians were more accepting of HMDs for immediate alerts. Both groups prioritized shared screens for critical and retrospective data, underscoring team coordination’s importance.

### Static Prototyping

The initial system design was developed using Figma, a cloud-based collaborative graphic design and user interface prototyping software. The interface was structured around role-specific needs and PALS guidelines. Through internal testing and iterative refinement, the focus was on optimizing readability, functionality, and clarity, ensuring the prototype was ready for end user evaluation.

#### Mobile Tablet App Design

**Figure 4.**
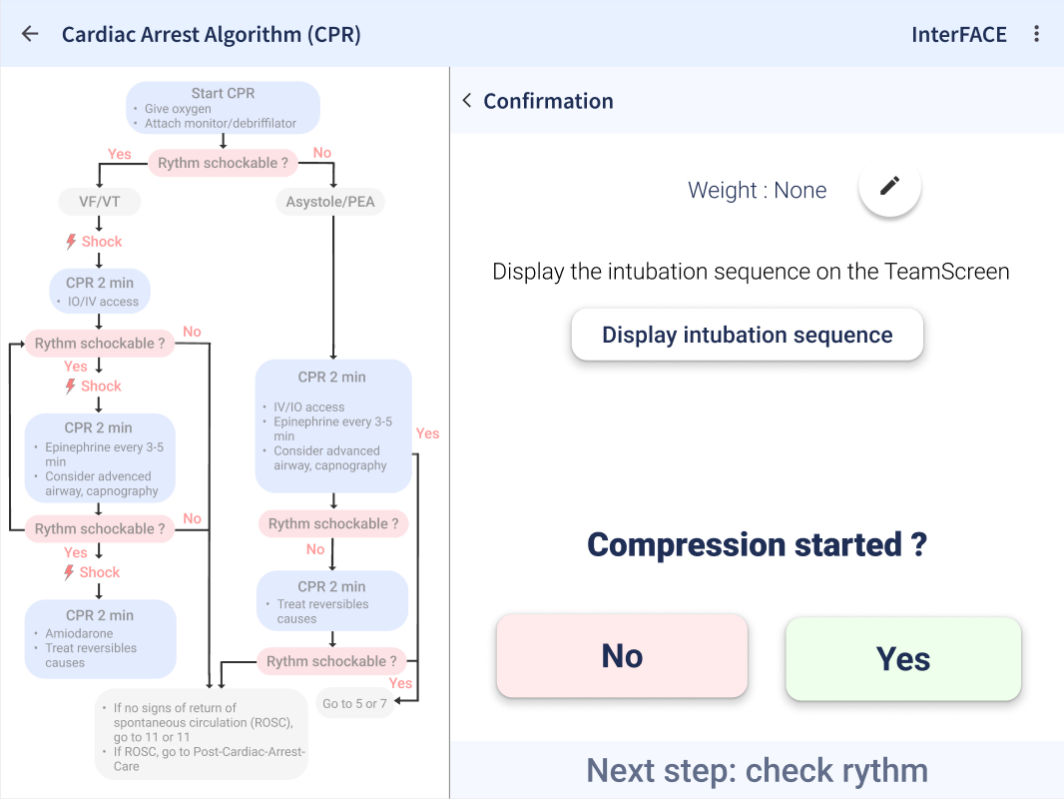
Screenshot of the guiding pad tablet app. This screenshot illustrates the guiding pad interface, with the left panel displaying the cardiac arrest algorithm as a visual workflow guide and the right panel presenting an interactive confirmation prompt to ensure adherence to critical steps, such as verifying chest compressions before proceeding.

The Guiding Pad tablet app was designed based on user interaction and cognitive workload analysis, resulting in a 2-zone interface (Figure 4). The left zone supports advanced interactions, such as navigating PALS algorithms, accessing clinical data, and adjusting to evolving patient conditions, while the right zone focuses on primary task execution, offering real-time, context-sensitive prompts aligned with PALS algorithms.

#### Team Screen Design

**Figure 5.**
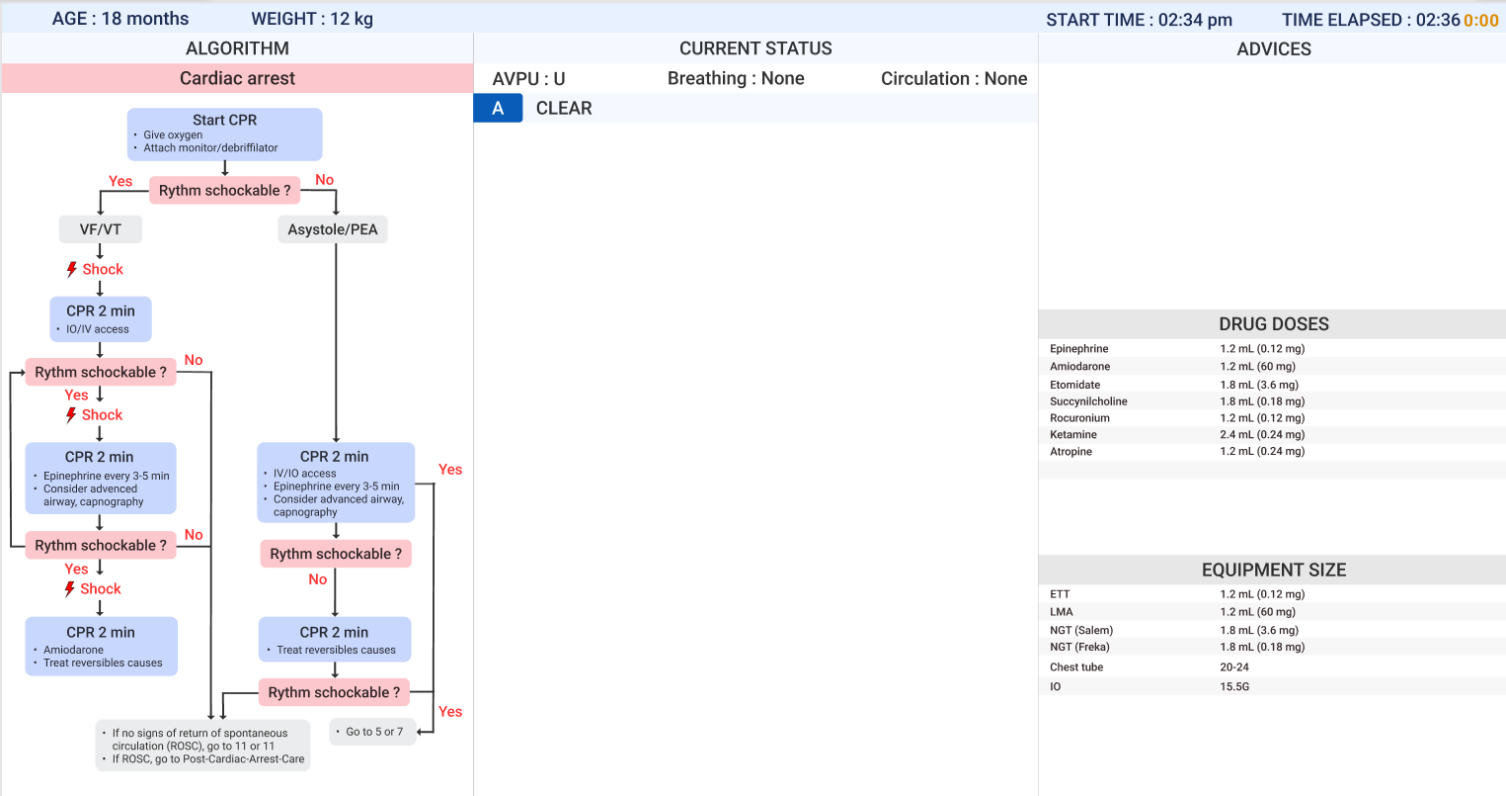
Early design for TeamScreen interface for PALS Cardiac arrest algorithm. Algorithm panel: cardiac arrest algorithm flowchart. Current status panel: progression in the ABCDE evaluation. Advices panel: advice for the current resuscitation step and information about drug doses and equipment size.

The team-wide display was organized into 3 functional sections to enhance situational awareness, prioritize actions, and streamline information retrieval (Figure 5). The left section visually represents the full PALS algorithm, enabling the team to track progress and anticipate steps. The center section highlights current required actions, ensuring team-wide awareness. The right section provides rapid access to critical drug information and ABCDE assessment results, minimizing workflow disruptions.

### AR HMD Design

**Figure 6.**
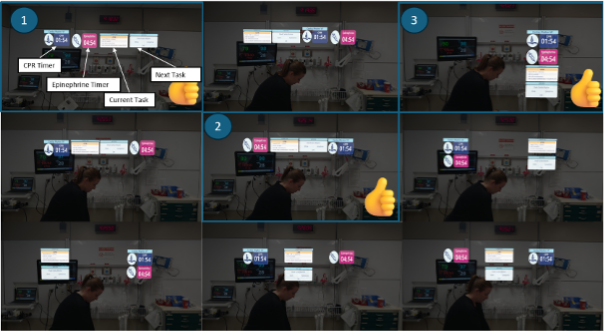
Static layout propositions for the augmented reality (AR) head-mounted display (HMD) of the team leader.

Based on the information prioritized during the initial design phase, several static layout prototypes were developed to explore different ways of presenting content within the AR display for the team leader (Figure 6). These mock-ups aimed to evaluate the most effective positioning of information in the field of view, with a focus on readability, prioritization of critical data, and minimizing cognitive load. Particular attention was paid to key AR design heuristics, including peripheral clarity (ensuring that essential information remains accessible even when not in central vision) and anchoring consistency (maintaining spatial predictability of interface elements).

### Dynamic Prototyping

#### Mobile Tablet App and Team Screen

**Figure 7.**
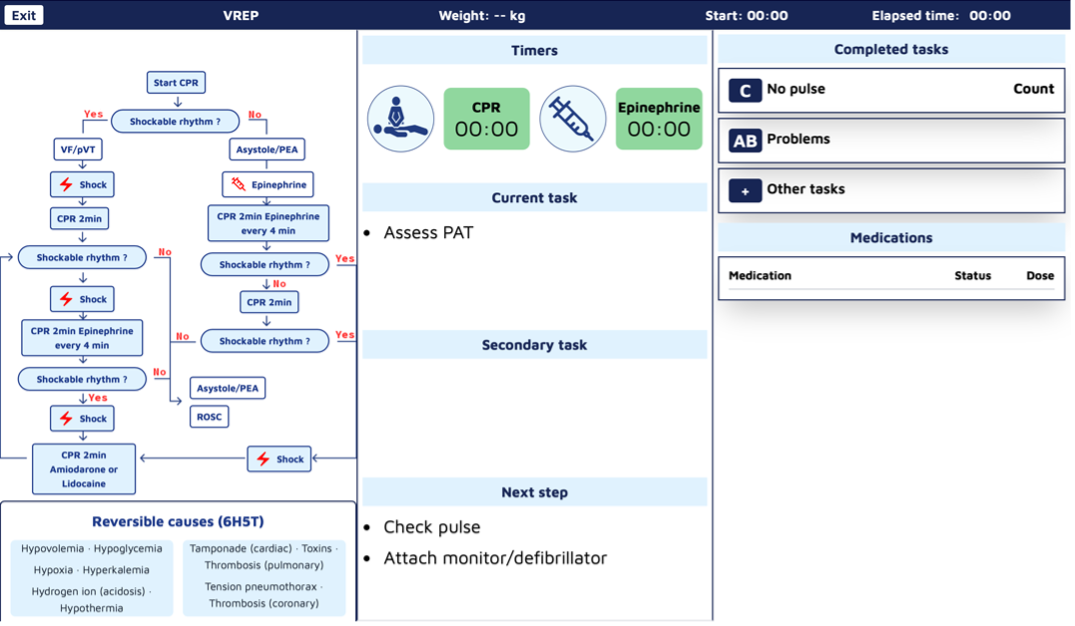
Screenshot of the TeamScreen. This screenshot depicts the interface for cardiac arrest management, comprising multiple panels. Left panel: pediatric advanced life support (PALS) cardiac arrest algorithm detailing steps for VF/pVT and asystole/PEA, including emergency drugs and shock decisions. Center panel: visual representation of the patient with cardiopulmonary resuscitation (CPR) and epinephrine timers, current tasks, secondary tasks, and next steps. Right panel: completed tasks log with categories (no pulse, AB problems, other tasks) and medication dosing section. Bottom panel: reversible causes (6H5T). AB : airway/breathing; PEA: pulseless electrical activity; VF: ventricular fibrillation; pVT: pulseless ventricular tachycardia.

Dynamic prototypes, developed in Figma, incorporated interactive elements simulating structured resuscitation workflows, task progression, and patient data visualization (Figure 7). A dozen weekly reviews with pediatric emergency and intensive care physicians, nurses, and system developers gathered qualitative feedback on information hierarchy, interaction efficiency, role-specific views, and guidance clarity. This ensured critical information was accessible, key actions required minimal steps, interfaces were tailored to roles, and system prompts supported clinical decision-making effectively.

User feedback drove key adjustments and interaction logic. The Guiding Pad adopted a 2-zone layout with the left zone for advanced interactions and the right zone for primary task execution and information access. The TeamScreen featured algorithm visualization on the left, prioritized actions in the center, and real-time ABCDE assessment and drug data on the right. Interaction flows were streamlined for seamless transitions between algorithm steps, enhancing usability and clinical alignment.

### AR HMD

In the dynamic prototyping phase, the static layouts were implemented within a functional AR environment to evaluate their behavior under real-time conditions. This phase focused on assessing how interface elements responded to user movement, task switching, and environmental complexity. A particular emphasis was placed on occlusion management, ensuring that critical real-world visual cues—such as team members, medical devices, and patient access—remained unobstructed by virtual overlays. The prototypes were tested using simulated clinical scenarios, allowing the team to refine spatial placement, interaction timing, and adaptive content behaviors based on contextual relevance and physical positioning. These evaluations informed iterative adjustments to optimize both usability and clinical safety in dynamic, high-pressure environments.

### Usability Testing of Individual Components

#### Guiding Pad

The Guiding pad usability test showed overall effectiveness, with the 15 participating nurses demonstrating ease of use during 27 tasks. Of these, 21 tasks achieved a 100% completion rate, while the remaining six ranged from 67% to 93% completion, indicating areas for refinement. The most frequently encountered issue involved advanced interactions, such as selecting the appropriate endotracheal tube size and insertion depth. Some participants were unaware that default values had been preselected. Another commonly failed task was the manual prescription of drugs; since the system automatically prescribed certain drugs, participants were sometimes uncertain about the required actions. Tasks completion times varied significantly, ranging from 2.93 seconds to 33.53 seconds on average, reflecting differences in task complexity.

#### TeamScreen

The TeamScreen evaluation, conducted with 5 clinicians and 15 nurses, highlighted the clear organization and meaningful presentation of information, with an 81% overall task success rate, reflecting participants’ ability to locate and interpret essential data under time pressure. Interface elements like shock logs, intubation timestamps, and reversible‐causes guidance were clear and easy to retrieve, but challenges in distinguishing ongoing CPR status and impending interventions (eg, epinephrine administration) highlighted the need for more intuitive visual structuring or prompts in high-density regions, as participants reported confusion over critical updates. Tasks completion times varied significantly, ranging from 2.35 seconds to 18.45 seconds on average, reflecting differences in task complexity.

#### AR HMD

In the usability evaluation of the AR HMD with 5 pediatric emergency physicians and 5 participating nurses participating, the system was found to be intuitive and efficient. Both the team leaders and medication nurses reported “Good” and “Excellent” SUS scores, respectively, with medication nurses benefiting from clear, task-specific prompts. The UEQ results indicated high attractiveness and positive evaluations in pragmatic qualities (intuitiveness, efficiency, and dependability), facilitating smooth task execution, and hedonic qualities (stimulation and novelty). The TAM confirmed high acceptance, with users perceiving the system as useful and easy to use during resuscitation scenarios.

### Informatics and System Architecture Development With Final Integration

The InterFACE system was designed as a real-time digital support tool for pediatric resuscitation, following a task tree model akin to a business process modeling notation (BPMN) [13], with each PALS algorithm step represented as a cursor (open, in progress, completed, or canceled) to support sequential execution and decision-driven adjustments based on clinical inputs. The system comprises a backend microservice (emergency-guideline-service) and role-tailored frontend applications. The backend, built as a Spring Boot microservice [14], handles business logic via a Representational State Transfer (REST) application programming interface (API) for data exchange and a server-sent events (SSE) API for real-time updates, following a domain-driven design approach with a root aggregate managing PALS algorithms, drugs, and clinical observations. An event-driven architecture [15] with event sourcing (ES) ensures data consistency and low latency, logging events in MongoDB (version 8.0) for real-time broadcasting via SSE, without requiring continuous polling.

**Figure 8.**
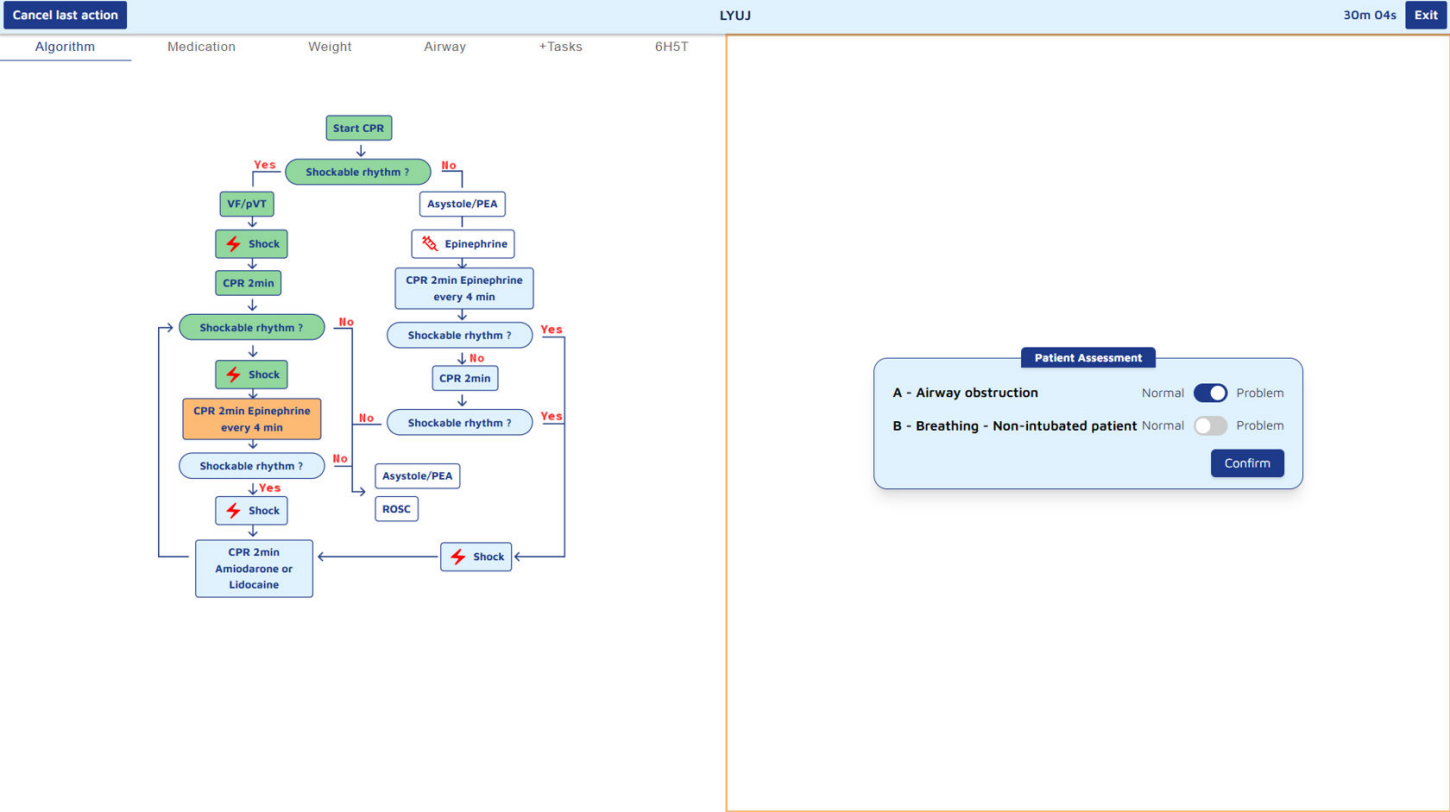
Implemented version of the guiding pad. The left side shows initially the CPR algorithm but enables also the access to advanced interaction such as medication prescription, weight input, airways documentation, and reversible causes documentation. The right part of the screen is dedicated to documenting the main tasks of the current step of the algorithm.

**Figure 9.**
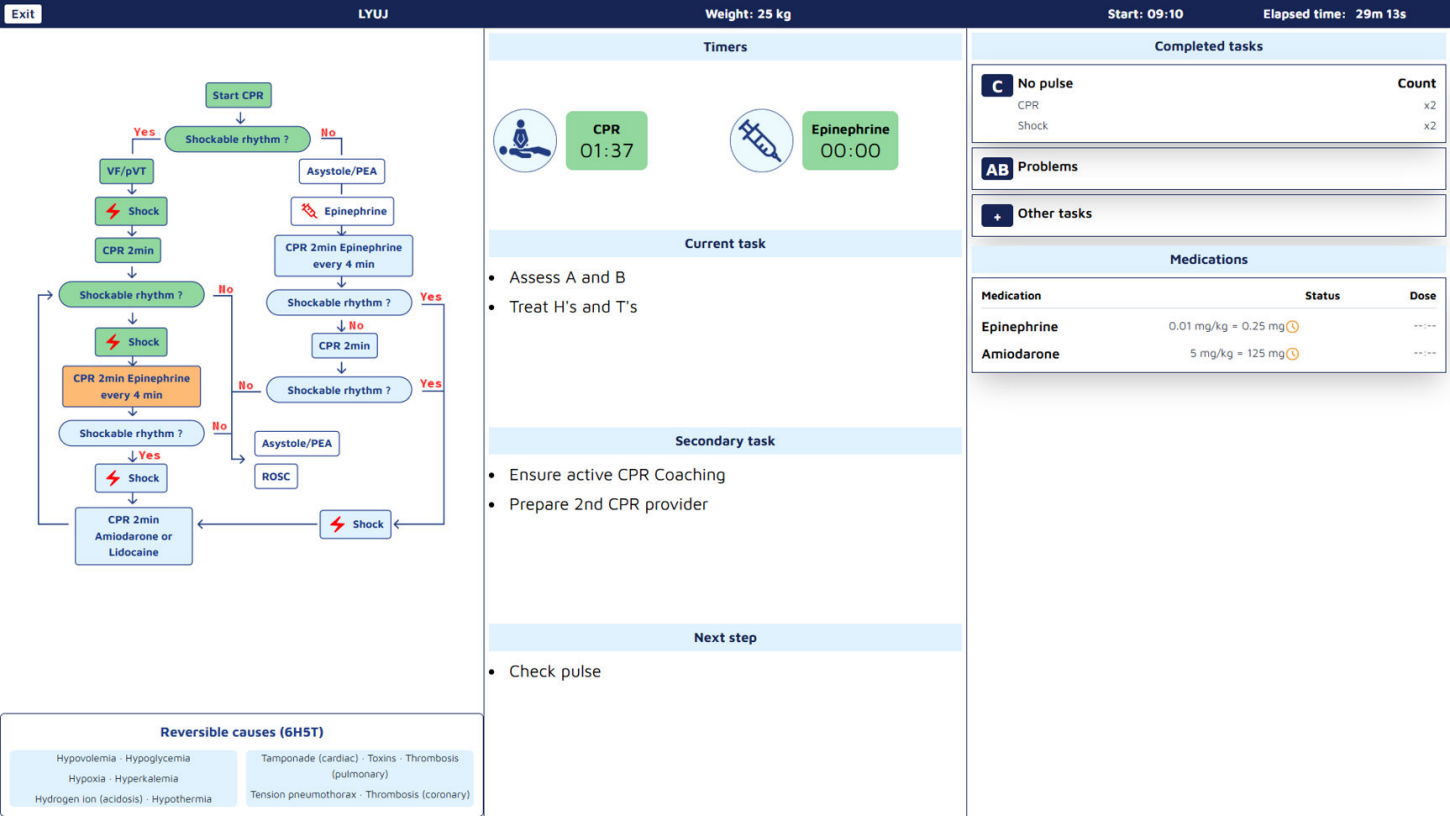
Implemented version of the TeamScreen. The left part contains the cardiopulmonary resuscitation algorithm showing the executed steps in green and the current one in orange. Underneath, we found the reversible causes. The middle column displays the most important information. Two counters indicate the cardiopulmonary resuscitation remaining time and the time to epinephrine administration. The middle part displays the current primary and secondary tasks, and the last part is dedicated to the next step. Finally, the right column provides information about the ABCDE assessment as well as a view on the administered medication.

**Figure 10.**
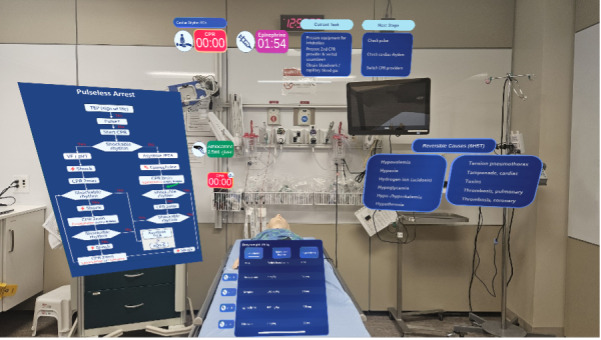
Vision of the team leader in the augmented reality (AR) display. The same information displayed in TeamScreen, such as the algorithm, the reversible causes, the current and next tasks, as well as the counter, is available on the field of vision.

Frontend modules, developed in Angular 17 JavaScript framework, include the Guiding Pad user interface (UI) for the scribe (supporting documentation, algorithm adjustments, and both sending commands and listening backend events; Figure 8), the TeamScreen UI for the team (displaying algorithm steps, prompts, and parameters in read-only mode while passively receiving real-time updates via SSE; Figure 9), and the AR HMD UI for the medication nurse and team leader (enabling hands-free data access; Figures10  11). Event sourcing transmits incremental changes to frontend clients—reducing data load by avoiding the need to send the entire state—with events timestamped and logged in MongoDB for full process reconstruction and auditability. For deployment, the backend runs as a Docker container, frontends are served via an NGINX server, and a short code identifies each resuscitation process, supporting concurrent sessions within the hospital network and remote guidance, allowing experts to assist or lead resuscitations from a distance. However, for performance optimization, real-time data is stored in memory and not persisted after server shutdown, as this proof-of-concept targets simulated patients in future studies, without authentication.

**Figure 11.**
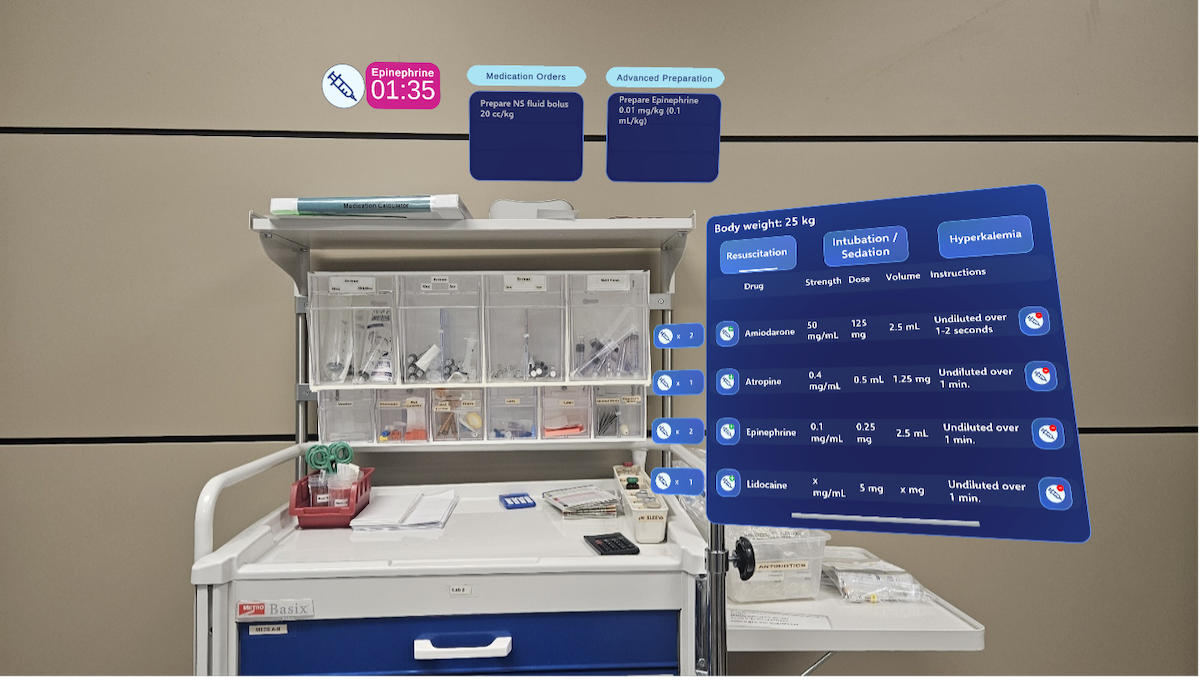
Final augmented reality (AR) head-mounted display (HMD) interface for the medication nurse showing role-specific information, such as drug doses, timers, and administration prompts.

## Discussion

### InterFACE System as a Cognitive Aid in Pediatric Resuscitation

InterFACE represents an advancement in digital cognitive aids for pediatric resuscitation, as evidenced by its design and evaluation through simulation-based approaches. The system integrates AR HMDs, tablets, and large displays within the resuscitation room, aiming to enhance shared cognition and temporal coordination among teams. This support is intended to ensure adherence to PALS guidelines, manage cognitive workload, and augment, rather than replace, clinical judgment, addressing key needs in high-stakes emergency situations. Although the personas themselves were not directly evaluated in the usability testing phase, they provided the conceptual scaffolding for the system’s architecture. By structuring the distribution of information and interaction logic around role-specific personas, the subsequent prototypes and evaluations could directly test whether these design assumptions translated into improved usability, workflow fit, and team coordination.

### Comparison With Existing Digital Resuscitation Tools

Several digital tools have been developed to support pediatric resuscitation, focusing on improving guideline adherence, reducing cognitive overload, and enhancing coordination. Previous research, such as studies on HMDs [4,16], team situation displays [17,18], mobile apps for medication administration [7,8], CPR corrective feedback devices [19,20], and tablet-based resuscitation guidance tools [5,6], highlights their benefits in specific areas. However, these technologies often lack comprehensive team-wide information sharing, role-specific guidance, and real-time adaptability to evolving clinical situations. InterFACE addresses these limitations by synchronizing multiple role-specific interfaces through an event-driven architecture with event sourcing, ensuring continuous real-time, context-adaptive updates without manual intervention. This facilitates seamless communication and coordination. Compared to PediAppRREST [6] and similar tools, InterFACE stands out by offering synchronous, role-specific guidance across multiple interfaces, including immersive AR displays, and by leveraging an event-driven architecture to ensure consistent, low-latency updates across the care team. This enables shared situation awareness and real-time decision support that is both context-sensitive and visually anchored in the physical care environment.

### Advancing Resuscitation Support With Mixed Reality

AR has shown promise in both resuscitation training and real-time procedural support. A recent systematic review highlighted the potential of AR in these areas ([21]). For example, an AR CPR feedback system provided real-time visual feedback on chest compression quality, significantly improving performance with compliance rates increasing from 18%‐21% to 87%‐90% ([16]). However, such devices are limited to specific tasks, like compression feedback, and do not offer comprehensive guidance for the entire resuscitation process. AR extends beyond AR by offering spatially anchored, interactive digital elements that provide hands-free, dynamic, and context-aware prompts. This technology enables a more holistic approach to resuscitation support. Via AR HMDs, InterFACE delivers real-time, role-specific decision support for critical tasks such as airway management, drug administration, and adherence to PALS algorithms. By providing a shared digital platform, InterFACE enables seamless team coordination to support the delivery of high-quality CPR.

While AR and virtual reality have been explored for resuscitation training, demonstrating potential in skill acquisition and knowledge retention, current evidence does not consistently show their superiority over traditional training methods [21]. This inconsistency limits their direct application in clinical settings. InterFACE addresses this gap by transitioning from static training models to real time, adaptive clinical support. By structuring digital support around team coordination and integrating directly into emergency workflows, InterFACE aims to optimize resuscitation performance beyond what is achievable through simulation alone, ultimately improving patient outcomes.

### Technical Contributions and Innovations

InterFACE is built on a microservices architecture to structure critical care workflows effectively, with a key innovation being its event-driven model using SSE for real-time updates, minimizing latency, reducing unnecessary data polling, and enhancing scalability in time-sensitive settings. It replaces BPMN-based execution with a flexible task-tree model, acknowledging the dynamic, nonsequential nature of resuscitation workflows in high-intensity environments.

### Challenges and Limitations

Several limitations must be acknowledged. First, the current evaluation focused exclusively on usability and acceptability in simulated environments and did not assess clinical outcomes in real resuscitation cases. While simulations provide a controlled setting to test usability and workflow integration, they cannot fully reproduce the stress, variability, and unpredictability of real emergencies. This may limit the generalizability of the findings. To mitigate this, scenarios were designed to be as realistic as possible, involving experienced clinicians. Future research should include randomized controlled trials in clinical settings.

Second, the complexity of pediatric resuscitation inherently increases cognitive load, particularly given the variability in patient age, size, and drug dosing. ([22]). Balancing comprehensiveness with simplicity in the interface was challenging. Iterative prototyping and multiple rounds of user feedback were used to refine the information hierarchy, but residual risks of information overload may persist. Future versions should explore adaptive content delivery and integration with patient-specific data.

Third, technical constraints limited system integration. The current prototype did not connect with live patient monitors or hospital information systems, which prevented inclusion of real-time vital signs—an item highly requested during the Delphi study. This may have reduced ecological validity. To mitigate this, placeholders and manual data inputs were provided to simulate the functionality. Future iterations should incorporate interoperability standards such as Fast Healthcare Interoperability Resources (FHIR) to enable seamless integration.

Fourth, the system was deployed in a single-instance Docker server without load balancing, restricting scalability for broader trials or multi-site use. Although this setup was sufficient for proof-of-concept testing, it may limit performance in high-demand contexts. Cloud-based deployment with redundancy and load balancing should be considered for larger clinical implementations.

In addition, the current version of InterFACE does not include core CPR quality metrics, such as compression-to-ventilation ratio, ventilation rate and volume, or end-tidal CO₂, all of which are crucial indicators of resuscitation performance and highly relevant for team leader oversight. This omission reflects the early-stage focus on workflow coordination and feasibility in simulation contexts, as well as technical constraints around integration with monitor data and CPR feedback devices. Future iterations of the system should prioritize the incorporation of these metrics through integration with patient monitors, compression sensors, and ETCO₂ waveforms, in order to provide the team leader with a more comprehensive, real-time view of resuscitation quality.

Fifth, some participants reported discomfort or distraction during prolonged use of AR HMDs. While refinements prioritized concise alerts and minimized unnecessary overlays, usability in extended real-world resuscitations may be affected. Gesture-based or multimodal interactions may improve user comfort in future versions.

Taken together, these limitations emphasize the need for further development, technical integration, and large-scale clinical validation before InterFACE can be widely adopted in routine pediatric resuscitation.

### Future Directions

Future work should prioritize clinical validation through randomized controlled trials, assessing impact on guideline adherence, response times, and error reduction across different hospital settings. Artificial intelligence–powered decision support, leveraging machine learning on large-scale CPR data, could offer predictive guidance by dynamically adapting to patient-specific factors, historical resuscitation patterns, and evolving clinical conditions. These advancements may reduce clinicians’ cognitive burden load while enhancing decision-making in time-sensitive situations. Scalability and multisite deployment, supported by cloud-based architectures with load balancing, are essential for broader adoption. Finally, integration with hospital information systems via FHIR standards would enable seamless data exchange with electronic health records and medical device data streams, positioning InterFACE as a transformative tool in pediatric resuscitation.

### Conclusion

This study provides a high-level overview of the user-centered, mixed methods process used to design and formatively evaluate InterFACE, an integrated augmented-reality decision support system for pediatric resuscitation. Usability testing across tablets, wall-mounted displays, and AR head-mounted devices demonstrated good acceptance, feasibility, and perceived usefulness in simulated scenarios. While clinical outcomes were not assessed, the results indicate that InterFACE can enhance team coordination and adherence to pediatric resuscitation guidelines in a controlled environment. These findings support the feasibility of deploying multimodal, AR-enhanced cognitive aids and provide a blueprint for further clinical evaluation.

## Abbreviations

AHA: American Heart Association
API: application programming interface
AR: augmented reality
BPMN: business process modeling notation
CPR: cardiopulmonary resuscitation
ES: event sourcing
FHIR: Fast Healthcare Interoperability Resources
HMD: head-mounted display
InterFACE: Interconnected and Focused Mobile Applications on Patient Care Environment
PALS: pediatric advanced life support
PSSUQ: Post-Study System Usability Questionnaire
REST: Representational State Transfer
SSE: server-sent event
SUS: System Usability Scale
TAM: Technology Acceptance Model
UEQ: User Experience Questionnaire
UI: user interface
UX: user experience
